# Machine learning approaches to study the structure-activity relationships of LpxC inhibitors

**DOI:** 10.17179/excli2023-6356

**Published:** 2023-09-05

**Authors:** Tianshi Yu, Li Chuin Chong, Chanin Nantasenamat, Nuttapat Anuwongcharoen, Theeraphon Piacham

**Affiliations:** 1 Center of Data Mining and Biomedical Informatics, Faculty of Medical Technology, Mahidol University, Bangkok, Thailand; 2 Beykoz Institute of Life Sciences and Biotechnology, Bezmialem Vakif University, Beykoz, Istanbul, Türkiye; 3 Streamlit Open Source, Snowflake Inc., San Mateo, California 94402, United States; 4 Department of Clinical Microbiology and Applied Technology, Faculty of Medical Technology, Mahidol University, Bangkok, Thailand

**Keywords:** antimicrobial resistance, LpxC, QSAR, machine learning, cheminformatics, activity cliff, chemotype

## Abstract

Antimicrobial resistance (AMR) has emerged as one of the global threats to human health in the 21st century. Drug discovery of inhibitors against novel targets rather than conventional bacterial targets has been considered an inevitable strategy for the growing threat of AMR infections. In this study, we applied quantitative structure-activity relationship (QSAR) modeling to the LpxC inhibitors to predict the inhibitory activity. In addition, we performed various cheminformatics analysis consisting of the exploration of the chemical space, identification of chemotypes, performing structure-activity landscape and activity cliffs as well as construction of the Structure-Activity Similarity (SAS) map. We built a total of 24 QSAR classification models using PubChem and MACCS fingerprint with 12 various machine learning algorithms. The best model with PubChem fingerprint is the Extremely Gradient Boost model (accuracy on the training set: 0.937; accuracy on the 10-fold cross-validation set: 0.795; accuracy on the test set: 0.799). Furthermore, it was found that the best model using the MACCS fingerprint was the Random Forest model (accuracy on the training set: 0.955; accuracy on the 10-fold cross-validation set: 0.803; accuracy on the test set: 0.785). In addition, we have identified eight consensus activity cliff generators that are highly informative for further SAR investigations. It is hoped that findings presented herein can provide guidance for further lead optimization of LpxC inhibitors.

## Introduction

The rampant use of antibiotics in human medicine and animal husbandry has led to the emergence of multidrug-resistant (MDR) pathogenic bacteria, which poses a growing threat to global public health. Amongst all antibiotics, about 70 % of pathogenic bacteria have developed resistance to at least one antibiotic (Bush and Bradford, 2016[2]). New antibiotics directed at novel targets are urgently needed to overcome resistance to existing antibiotic classes. The most important features distinguishing Gram-negative organisms from Gram-positive organisms, is outer membrane. As a significant challenge to antimicrobial agents due to its remarkable capabilities to restrict the access of small molecule drugs to the periplasmic space, the outer membrane of Gram-negative bacteria has been exploited for its biogenesis pathways to find new antibiotic targets. Among the various checkpoint enzymes that are responsible for outer membrane assembly and lipid A synthesis, the UDP-3-O-(R-3-hydroxymyristoyl)-N-acetylglucosamine deacetylase, encoded by LpxC, is a critically important enzyme in the lipid A biosynthetic pathway, and is considered as a novel antibiotic target for the containment of MDR Gram-negative bacteria. LpxC is a single-copy gene conserved in all Gram-negative bacteria. The UDP-3-O-(R-3-hydroxyacyl)-N-acetylglucosamine deacetylase (LpxC) is a zinc ion-dependent enzyme catalyzing the first irreversible step of lipid A (as hydrophobic membrane anchor of lipopolysaccharide (LPS) which is critical for cell viability) biosynthesis. Unlike human proteins, LpxC does not share any sequence or structural homology. Therefore, it may become a novel target for the new drugs against MDR Gram-negative bacteria (Erwin, 2016[6]; Onishi et al., 1996[16]; Young et al., 1995[26]).

The drug discovery of LpxC inhibitors dated back to the 1980s. To date, numerous LpxC inhibitors have been developed, including ACHN-975, which has entered clinical trials. On the one hand, it is a selective LpxC inhibitor with a sub-nanomolar potency, on the other hand, it is a potent compound covering a broad spectrum of Gram-negative bacteria. However, clinical trial phase I was discontinued due to local inflammation at the injection site (ClinicalTrials.gov Identifier: NCT 01597947) and cardiovascular toxicities in mice models. Considering the structural perspective, most of the developed LpxC inhibitors contain a hydroxamate group as the chelating 'warhead' targeting the catalytic zinc ion of LpxC. However, recent studies have explored non-hydroxamate-containing molecules, such as TP0586532 and 2-(1S-hydroxyethyl)-imidazole derivatives (Fujita et al., 2022[8]; Yamada et al., 2020[24]). These newly developed non-hydroxamate-containing molecules demonstrate potent inhibitory activities against MDR *P. aeruginosa *and* Enterobacteriaceae*. Due to the critical role of LpxC as a lucrative antibacterial target, and the paucity of successful FDA-approved LpxC inhibitors, there is an urgent need to further explore the structure-activity relationships of LpxC inhibitors and develop more optimal inhibitors. 

QSAR/QSPR is a kind of mathematical model to investigate quantitative structure-activity/property relationship of chemical entities. There are two fundamental logical principles underlying QSAR/QSPR: (i) compound structure dictates its bioactivity and (ii) structurally similar compounds demonstrate similar bioactivities or properties (Tropsha, 2010[23]). There are two kinds of QSAR/QSPR based on the tasks: classification QSAR/ QSPR model and regression QSAR/QSPR model. The former aims to predict the bioactivity classes of compounds, such as active/inactive class of enzyme inhibitors, agonist/antagonist category of biological receptors; while the latter aims to predict the detailed values of compounds, such as pIC_50_ of DNA gyrase inhibitors, melting point of certain biomaterials. At this moment, QSAR/QSPR has become a practical powerful tool for computational drug discovery. In addition to drug discovery, they are also widely used in organic/inorganic chemistry, material science, chemical biology, forensic toxicology, and even environmental protection. Due to its wide spectrum of utilities, the OECD countries have now already established principles for QSAR modeling consisting of five rules: defined endpoint, unambiguous algorithms, defined applicability domain, modeling validation, and mechanistic interpretation to standardize the application of QSAR/QSPR modeling. This involves all steps of modeling process: data collection, data preprocessing, data splitting, machine learning modeling process, validation of the model, and mechanistic interpretation of feature importance (Fjodorova et al., 2008[7]; Piir et al., 2018[17]; Tropsha, 2010[23]).

In this study, we have performed a QSAR modeling study for LpxC inhibitors from the ChEMBL database to predict inhibitory bioactivities. In addition, we have visualized and analyzed chemical space, structure-activity landscape, and activity cliffs within the datasets. All the modeling and findings in the study can serve further lead optimization for more LpxC inhibitors.

## Materials and Methods

**Figure 1:**
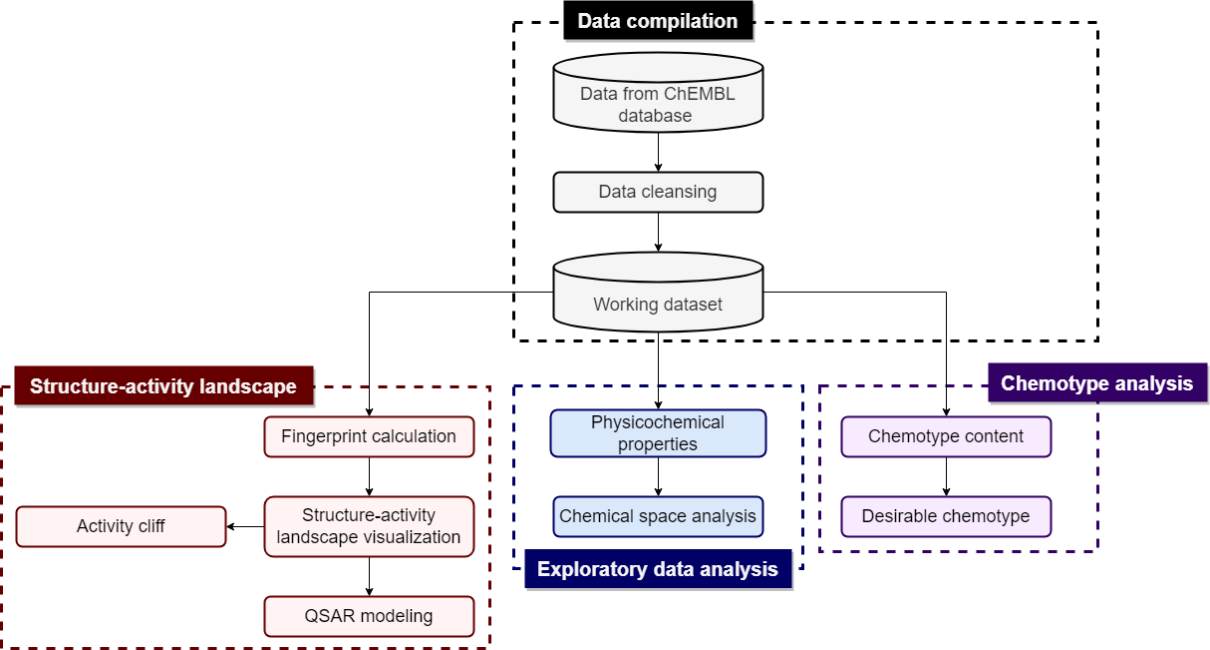
Methodological workflow employed for this study. Cylinders denote the data sets and rectangles denote the processes.

The methodology adopted in this computational study is summarized in Figure 1(Fig. 1). The study design consists of data compilation, exploratory data analysis, structure-activity landscape, and chemotype analysis.

### Data compilation

Data sets of inhibitors against LpxC (Target ID: CHEMBL 3855) employed in this study were retrieved from the ChEMBL 31 database. There was a total of 587 bioactivity data points with IC_50_ values for LpxC. The data set was then pre-processed by removing the redundant, unqualified, and missing data points, resulting in a working data set consisting of 491 compounds.

### Methodology overview

As the focus of this study is on the development of classification models of biological activity, the bioactivity data points of LpxC inhibitors were indicated by IC_50_ and further transformed to pIC_50_ by taking the negative logarithm to the base of 10.

The compounds with pIC_50_ values greater than 9 (pIC_50_ ⩾ 9, corresponding to an IC_50_ value of 1 nM) were categorized as potent. Those with pIC_50_ values ranging between 8 and 9 (9 > pIC_50_ ⩾ 8, corresponding to an IC_50_ value of 1 - 10 nM) were categorized as active whereas those with of less than 7 (pIC_50_ < 7, corresponding to an IC_50_ value of 100 nM) were categorized as inactive. Moreover, the intermediate bioactivity data with pIC_50_ values ranging between 7 and 8 were categorized as intermediate. 

### Molecular descriptor generation and calculation

The DataWarrior software (Sander et al., 2015[18]) was used to compute a total number of six descriptors on physicochemical properties associated with drug-likeness: molecular weight (MW), octanol-water partition coefficient (Log P), number of hydrogen bond acceptors (nHA), number of hydrogen bond donors (nHD), number of rotatable bonds (nRot) and topological polar surface area (TPSA). This chemical space analysis was performed on two groups of compounds, defined as group1 (potent and active classes, or pIC_50_ ⩾ 8) and group2 (intermediate and inactive classes, or pIC_50_ < 8).

### Univariate and multivariate analyses

As an exploratory data analysis, univariate statistical analysis was conducted to investigate the different patterns and trends of individual molecular descriptors between two groups of compounds using 6 descriptive statistical parameters: the minimum (Min), first quartile (Q1), median, mean, third quartile (Q3) and maximum (Max). In addition, statistical differences of descriptors among two groups of compounds were evaluated using the p-value obtained from Student's t-test.

Principal component analysis (PCA) as a dimensionality-reduction unsupervised machine learning method is executed to visualize the distribution patterns, overlapping of the molecules.

### Structure-activity relationship

Structure-activity relationship (SAR) is based on the idea that structure dictates activity, and molecules with similar structures demonstrate similar bioactivities. The publicly available structure-activity data of LpxC inhibitors provides an opportunity to mine SAR. The SAR landscape can be considered as a chemical space with an extra dimension of biological activity. Thus, in this study, structure-activity similarity (SAS) maps and structure-activity landscape index (SALI) values were used to visualize the structure-activity landscape and identify activity cliffs.

A SAS map is a tool for SAR analysis of compound data sets tested with one molecular target. The plot is a pairwise 2D plot of activity difference against structure similarity and consists of four quadrants: smooth regions of the SAR space, rough region of activity cliffs, nondescript region (i.e., low structural similarity and low activity similarity) as well as scaffold hopping region (low structural similarity but high activity similarity). Activity Landscape Plotter V.1, a webserver, is used to generate SAS maps by quantifying the activity cliffs (González-Medina et al., 2017[11]). SALI value is a pairwise measure between activity difference and structural difference for each pair of compounds and was calculated as Eq. 2, proposed by Guha and Van Drie (Guha, 2012[12]):


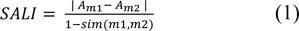


where A_m1_ and A_m2_ are the activities of molecule 1 (abbreviated as m1) and molecule 2 (abbreviated as m2) while sim (m1, m2) is referred to the similarity coefficient between two molecules (in this work computed with the PubChem and MACCS fingerprint). The SALI value increases with the possibility of the pair of compounds forming ACs. The values were mapped onto the SAS maps using a continuous color scale, ranging from green color (structurally most similar pairs) to red color (least similar pairs). In this study, the activity of molecules is represented by pIC_50_ values of molecules whilst similarity is represented by PubChem and MACCS fingerprint similarity.

The identification of AC is one of the main applications of activity landscape methods. The criterion of AC depends on two variables: fingerprint similarity and activity difference (Cruz-Monteagudo et al., 2014[5]; Stumpfe et al., 2019[20]). The threshold of activity difference is set to two magnitudes as default, which means that pIC_50_ level differences should be ≥ 2, and similarity set according to the SAS map statistics where mean+2 standard-deviation difference to be the threshold.

### Molecular descriptors generation

Molecular fingerprints are the representations of a complex form of molecular descriptors, which describe molecules in terms of their constitution, connectivity, and physicochemical properties. They are typically encoded by bit strings to characterize a given molecule. In this study, PubChem and MACCS fingerprints provided by the PaDEL package (Yap, 2011[25]) were used for modeling. The former contains 881 binary representations of the chemical structure fragments, and the latter contains 166 binary representations of the chemical structure fragments.

### Feature selection

A feature selection procedure was conducted to improve the accuracy of the QSAR model and to avoid overfitting. In this procedure, the correlation-based filter method was deployed: low-variance features (variance < 0.1), features with collinearity (correlation > 0.90) were removed, so that feature complexity is decreased.

### Data balancing and splitting

**Figure 2:**
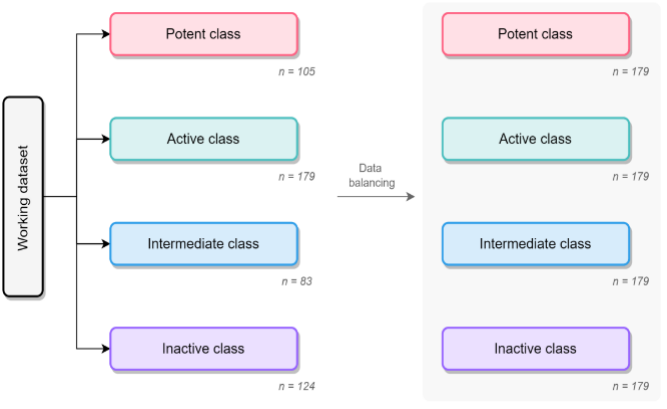
Comparison between working datasets and balanced datasets

The working dataset from the previous step of data cleansing was noticeably imbalanced between various bioactivity classes (*e.g.* the ratio of intermediate ligands to active ligands is more than two) as shown in Figure 7. To avoid any overfitting due to data imbalance, the datasets were then further balanced via the oversampling technique, which means the data are randomly duplicated in minority classes. After data balancing, the balanced datasets were subjected to further split into training and testing sets according to the ratio of 80:20. The changes of data before and after the data balancing process is illustrated in Figure 2(Fig. 2). 

### QSAR model construction

**Figure 3:**
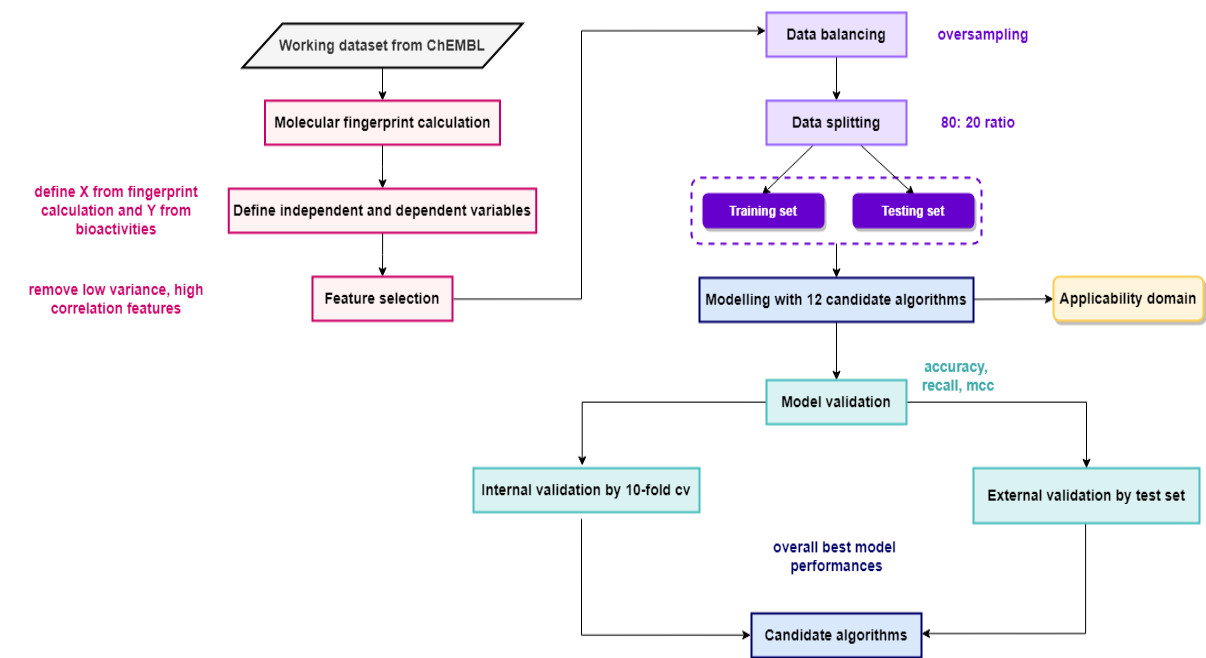
Workflow of the QSAR study. Different colors represent different procedures of QSAR modeling process: black for data collection and data cleansing, pink for molecular fingerprint calculation, purple for data balancing and splitting, blue for QSAR modeling, turquoise for model validation and yellow for determination of applicability domain

The QSAR models in this study are multiclass classification models with aims of predicting four bioactivities of LpxC inhibitors, namely potent class, active class, intermediate class, and inactive class. Hereby, to facilitate multiclass classification modeling, the one-vs-rest (OVR) approach is utilized. Shown in Figure 3(Fig. 3) is the workflow for QSAR modeling. To get the best model, 12 machine learning algorithms for classification have been employed independently for model construction, as shown in **Results & Discussion**. The performance for each model is evaluated and the algorithm yielding the best performance will be taken for downstream analysis.

### QSAR model validation

There are two aspects of QSAR model validation: internal validation and external validation.

### Internal validation

In this study, the balanced dataset was subjected to further split into training and testing sets according to the ratio of 80:20. Within the training set, a 10-fold cross-validation was performed to guarantee the robustness and reliability of the model. Briefly, the training data is divided into ten folds and used each fold for the internal validation while the rest nine folds are used to train the model. This process was repeated iteratively until all folds were used for validation.

### External validation

The prediction performance of the QSAR classification models was evaluated via three parameters, namely accuracy (ac), recall (re), and Matthew's correlation coefficient (MCC) (Chicco and Jurman, 2020[4]), which are defined by the following equations:


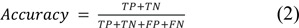



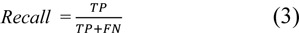



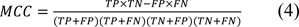


where TP, TN, FP, and FN denote true positive, true negative, false positive, false negative, individually. The high accurate model yields a high ac and re values (maximum of 1). A perfectly classified model yields a high MCC value, approaching 1, while low MCC value (minimum of -1) represents a perfect misclassification in the QSAR model.

### Applicability domain determination

The applicability domain (AD) of the QSAR models in this study are assessed by means of the principal component analysis (PCA) bounding box. This essentially entails comparing the chemical space of compounds from the training set with those from the test set via PCA analysis of scores plot. DataWarrior (Sander et al., 2015[18]) is used for AD determination by PCA.

As mentioned in the **Introduction** section, the OECD countries have established principles for QSAR modeling. The robustness of the QSAR models in this study are shown in Table 1(Tab. 1) in line with OECD criteria (Fjodorova et al., 2008[7]; Piir et al., 2018[17]; Tropsha, 2010[23]).

### Chemotype analysis

We conducted chemotype analysis to gain insights to the representative molecular scaffolds. In this study, we utilized Murcko scaffold approaches to conduct chemotype analysis. Murcko and Bemis dissect a molecule into four parts: ring systems, linkers, side chains, and the Murcko framework combinates ring systems and linkers in a given molecule (Bemis and Murcko, 1996[1]). In this study, Murcko scaffolds and cyclic skeleton systems are generated for LpxC inhibitors and compared by corresponding pIC_50_ levels, so that favorable, frequent, unfavorable scaffolds can be identified. DataWarrior (Sander et al., 2015[18]) is used for scaffold generation and analysis.

## Results

### Exploratory data analysis

A total number of 587 LpxC inhibitors were retrieved from the ChEMBL database. A working dataset of non-redundant compounds consisting of 491 LpxC inhibitors was obtained after pre-processing data, as summarized in Table 2(Tab. 2), and then subjected to further investigation. 

**Figure 4:**
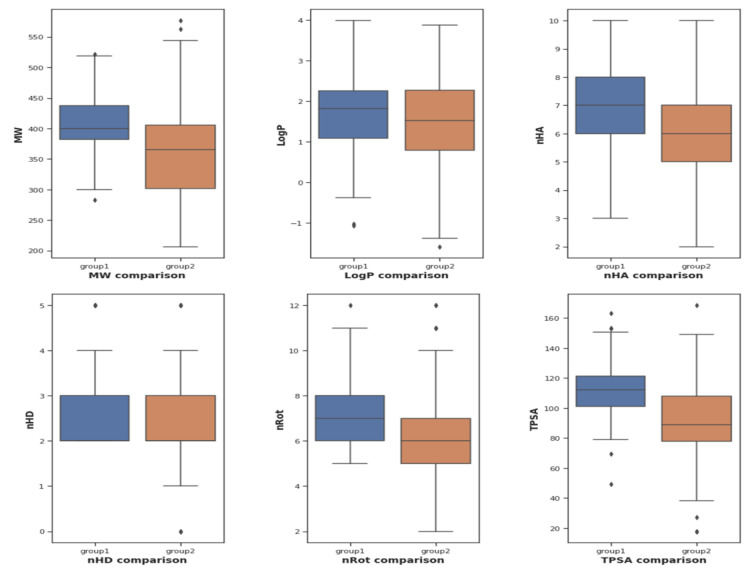
Box plot of physicochemical properties between group1 and group2 molecules of LpxC inhibitors. Group1 molecule, indicated potent and active groups, is represented with blue colour while group2 molecule, indicated intermediate and inactive groups, is represented with brown color.

To determine the different characteristics between two groups of molecules (group1: potent and active; group2: intermediate and inactive), an exploratory data analysis of six drug-likeness descriptors was performed via statistical analysis (Figure 4(Fig. 4) and Table 3(Tab. 3)). This analysis depicted that most LpxC inhibitors abide by drug-like properties according to Lipinski's rule of 5 and other drug likeness rules (Ghose et al., 1999[10]; Lipinski et al., 2001[14]; Muegge et al., 2001[15]). All the six properties demonstrated non-parametric distribution patterns, except for nHA and nRot. After the Mann-Whitney U test, MW, nHD and TPSA have p-values < 0.05, meaning that they demonstrate statistical significance. Since nHA and nRot properties abide by normal distribution, t-test is used for checking p-values. Both nHA and nRot demonstrate statistical significance with t-test. Generally, group1 molecules have higher MW, nHA, nHD, nRot and TPSA values than group2 molecules.

### Principal component analysis

**Figure 5:**
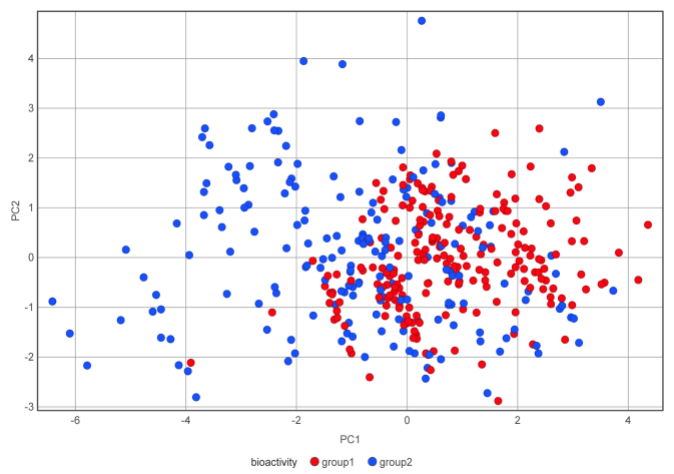
PCA for the six physiochemical properties of the LpxC inhibitors. Compounds from group1 and 2 are represented by red and blue dots, respectively.

PCA was applied to explore the chemical space of LpxC inhibitors as shown in Figure 5(Fig. 5). PCA plot with six physicochemical properties has shown that group1 molecules generally differ significantly in chemical space from group2 molecules. Group1 molecules occupy the concentrated area within the chemical space, mostly contained by group2 molecules. PCA plot has indicated that group1 molecules are less diverse than group2 molecules.

The eigenvalues of the six properties as shown in Table 4(Tab. 4) have revealed the three principal components contribute about 90% of the whole data sets. PC1 is primarily contributed by nHA (0.510) and MW (0.494), followed by TPSA (0.491), nRot (0.437). PC2 has the highest loadings by nHA (0.226) while LogP (-0.783) and nRot (-0.407) are the most significant negative contributors. PC3 has the most significant negative contributor nHD (-0.941).

### Structure-activity landscape (SAL) visualization

**Figure 6:**
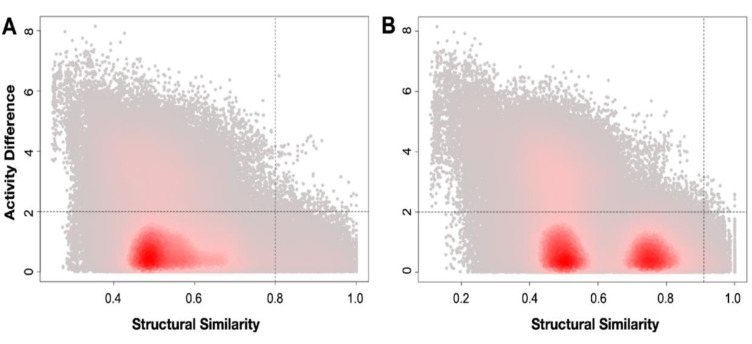
Structure-activity landscape (SAL) of LpxC inhibitors as visualized by the density SAS map. Panel A is the density SAS map using the PubChem fingerprint while B is for the MACCS fingerprint.

According to Table 5(Tab. 5), the mean and standard deviations of the fingerprint similarities are listed. As described in the methodology, the similarity criterion to define AC is set to mean+2 standard deviation, *i.e.*, 0.80 for PubChem fingerprint, 0.91 for MACCS fingerprint. The activity magnitude is set to two. The AC quadrants in Figure 6(Fig. 6) are both marginal and sparse, so that the existence of SAR discontinuities does not affect the overall SAL. This indicates the feasibility of building QSAR models using the fingerprints with the LpxC datasets.

### Quantitative structure-activity relationship (QSAR) modeling and validation

Both PubChem and MACCS fingerprints are selected in combination with 12 representative classification algorithms. Based on the model 1 performance metrics shown in Table 6(Tab. 6), Random Forest algorithm, also known as RF, provides the best performance with accuracy of 0.955 in the training set, 0.823 in the 10-fold cross validation set and 0.826 in the testing set. Following RF, other algorithms including Extra trees (ET), Extreme gradient boost (XGB), K-nearest neighbor (KNN), and Multilayer perceptron (MLP) provide equivalently good model performances. Whilst Naive Bayes (NB) algorithm is the least ranked algorithm. As shown in Supplementary Figure 1A, the test set falls within the training set of the PCA plot. For model 2 performance metrics (Table 7(Tab. 7)), RF and NB are also the best and worst performing algorithms, respectively. As shown in Supplementary Figure 1B, the test set falls within the training set of the PCA plot, as well.

### Activity cliff visualization

**Figure 7:**
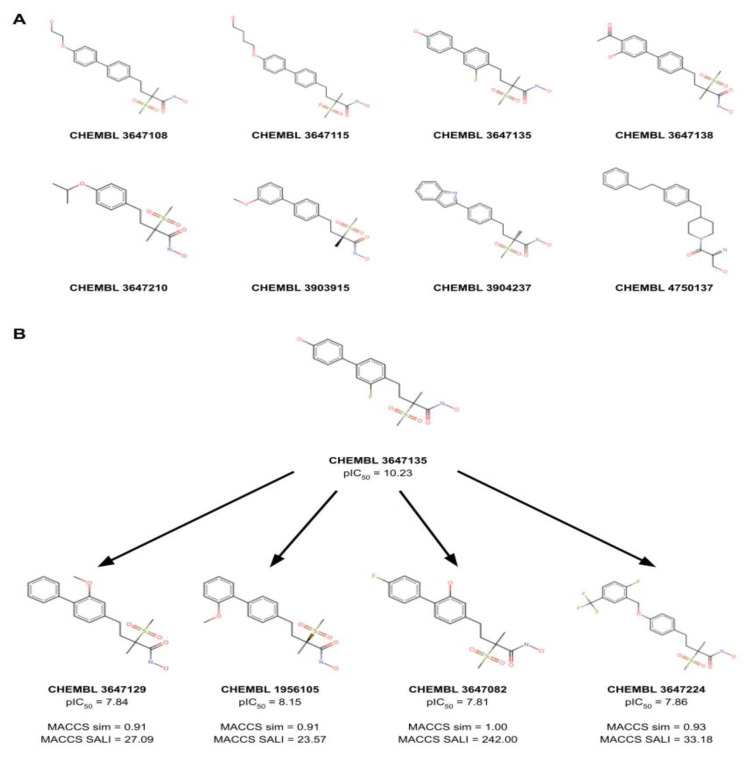
Activity cliff visualization. (A) All the eight common activity cliff generators and (B) representative activity cliffs that are formed with pairwise molecules

According to the threshold criteria listed in **Materials and Methods**, there are a total of 367 and 103 activity cliffs in PubChem and MACCS fingerprint, respectively. There are 82 common activity cliffs between these two fingerprint datasets. Amongst the activity cliffs, there are eight common activity cliff generators. Figure 7A&B(Fig. 7) depicts the chemical structure of all the eight common activity cliff generators and the representative activity cliffs that are formed with pairwise molecules, respectively. The existence of activity cliffs is detrimental to development of QSAR predictive models, nevertheless, this provides highly informative insights into the SAR of molecules for medicinal chemists (Cruz-Monteagudo et al., 2014[5]). These activity cliffs and activity cliff generators can provide important guidance to lead optimizations.

### Chemotype determination and chemotype analysis

Shown in Table 8(Tab. 8) is scaffold diversity amongst different subsets of LpxC molecules. Generally, molecules in group1 demonstrate lower scaffold diversity than molecules in group2. Therefore, there is an urgent need to find more novel scaffolds for LpxC inhibitors.

**Figure 8:**
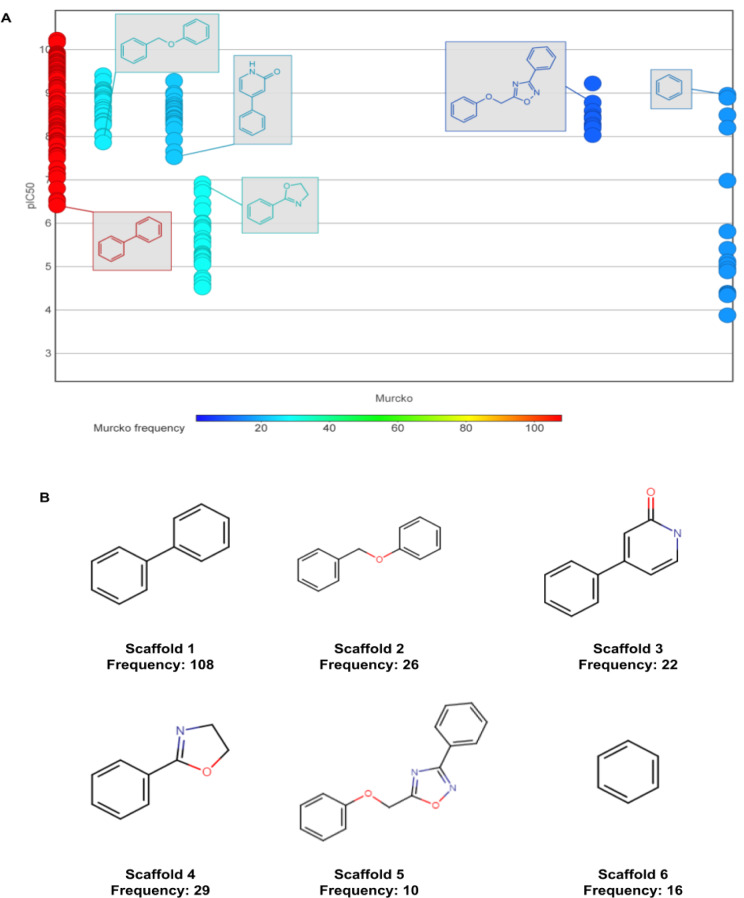
Chemotype analysis for LpxC inhibitors. (A) Scaffold (frequency ≥ 10) versus bioactivity plot and (B) Top six Murcko scaffolds visualization

In scaffold analysis, a total of six Murcko scaffolds (Ns) with frequency ≥ 10 were extracted as shown in Figure 8(Fig. 8). Scaffold 1, with frequency of 108, is biphenyl scaffold. Amongst these 108 molecules, nine of them are with pIC_50_ ≥ 8, even 9. All of them are the combinations of scaffold 1 and hydroxamic acid as the chelating moieties. Scaffold 2, with frequency of 26, is benzyloxy benzene. Scaffold 3, with frequency of 22, is 4-phenyl-1,2-dihydropyridin-2-one, Scaffold 4, with frequency of 29, is 2-phenyl-4,5-dihydro-1,3-oxazole. This scaffold is seen in early developed LpxC inhibitors in the 1990s, such as the L-573655, L-161240 by Merck company. Scaffold 5, with frequency of 10, is 5-(phenoxymethyl)-3-phenyl-1,2,4-oxadiazole. Scaffold 4, with frequency of 29, is 2-phenyl-4,5-dihydro-1,3-oxazole. This scaffold is seen in early developed LpxC inhibitors in the 1990s, such as the L-573655, L-161240 by Merck company. A series of scaffold 1 based analogs were designed and synthesized to optimize bioactivities. Scaffold 6, with frequency of 16, is benzene ring that is abundant in many newly developed LpxC inhibitors. The first one is ACHN-975 and is also the sole LpxC inhibitor that has entered clinical trials till date (Krause et al., 2019[13]). The benzene ring is addicted with a side chain of hydroxamic acid as the head, and on the para position linked to an aliphatic side chain with two triple bonds as the tail. Although clinical trials of ACHN-975 terminated due to tachycardia and hypotension side effects, the molecular scaffold is expected to generate more optimal lead molecules.

## Discussion

AMR is a rapidly growing concern in public health. There are many efforts of various explorations to overcome the challenge of AMR, including the development of direct-acting antibacterial against novel targets, drug-repurposing, antibiotic potentiators, anti-virulence approaches, immune modulators, etc. According to statistics, direct-acting antibacterial against novel targets account for the most significant projects (Theuretzbacher et al., 2020[21]). LpxC inhibitors are one of the most promising novel direct-acting antibacterials in the preclinical pipeline. Although there are ACHN-975 and RC-01 that have entered clinical trials, they both have been terminated due to safety issues. Based on the core structure of ACHN-975, there are some additional inhibitors, such as LpxC -289, LpxC -313 and LpxC -516 that have arisen attentions as they demonstrate potent LpxC inhibitory activities *in vitro* and better safety profiles, and LpxC-516 is the best (Krause et al., 2019[13]).

Like conventional bacterial targets, LpxC inhibitors will inevitably encounter resistance due to various mechanisms. The primary factor contributing to resistance to LpxC inhibitors is efflux pump in *P. aeruginosa*, to date. For *Enterobacteriaceae*, however, overexpression of efflux pumps has not been reported (Caughlan et al., 2012[3]; Tomaras et al., 2014[22]). As a novel target, there are no known resistance genes on mobile elements for LpxC. The only identified chromosomal point mutation of the cytosine 11 bp upstream (to adenine or guanine or deletion) of the LpxC start site resulted in elevated MICs for LpxC inhibitors. However, this mutation is relatively rare and occurs with a low frequency (Krause et al., 2019[13]). Previous study has proved the unique mechanism of resistance to LpxC inhibitors in *E. coli* by mutations of fabZ, a dehydratase in fatty acid biosynthesis and thrS, Thr-tRNA ligase through rebalancing bacterial cell homeostasis (Zeng et al., 2013[27]).

Apart from being direct-acting antibacterial, it is important to note that LpxC inhibitors can play the role of antibiotic potentiators by sensitizing bacteria to conventional antibiotics, as well (Erwin, 2016[6]). This has been demonstrated in animal models, where synergistic effects of PF-5081090 have been observed with polymyxin B nonapeptide in a mouse model of *P. aeruginosa* infection and synergy of both rifampin and vancomycin with LpxC inhibitors of *P. aeruginosa* and *K. pneumoniae* in mouse models (Erwin, 2016[6]).

Previous studies exploring the SAR of LpxC inhibitors have used a variety of methodologies. For instance, the group of Zuo performed 3D-QSAR studies with pyridone methyl sulfone hydroxamate molecules (Zuo et al., 2017[28]). There are additional studies focusing on 3D-QSAR with satisfactory model performance and validated by molecular dockings(Shiri et al., 2018[19]). The group of Ghasemi has devised a new methodology of QSAR using LpxC inhibitors by integrating interaction energies of molecular dynamics trajectories and QSAR modeling (Ghasemi et al., 2012[9]). In comparison with previous representative studies, this study uses conventional QSAR modeling approaches instead of 3D or 4D QSAR approaches, therefore, the conformational and 3D-structural aspects are not incorporated into the modeling process, which is a noticeable drawback in study design. On the other hand, the size of data sets that are compiled from the ChEMBL database turn out to be much bigger and more diverse, comprehensive. Therefore, the applicability domain of this study is broader. 

The significance of the study can be concluded by three aspects: First and foremost, the two QSAR models we built demonstrate robustness and reliability in performance, in line with OECD criteria (Fjodorova et al., 2008[7]). Both can be used as bioactivity predictors for potential new chemical entities. Besides, the activity cliffs and activity cliff generators identified in this study provide inspirational information for further lead optimization.

## Conclusions

AMR is one of the most serious global health threats globally of the late 20^th^ and 21^st ^century. Drug discovery of inhibitors against novel targets rather than conventional bacterial targets has been considered an inevitable strategy to address the growing threat of AMR infections. This study investigated the structure-activity relationship (SAR) of LpxC inhibitors using QSAR modeling and cheminformatics analysis. The best QSAR models built with the PubChem and MACCS fingerprint are using XGB and Random Forest algorithms, respectively. In addition, we have identified eight consensus activity cliff generators that provide highly informative insights on the SAR. It was found that scaffolds 2, 3 and 5 are favorable scaffolds while scaffold 4 is the unfavorable scaffold. In addition, scaffold 1 is the most prevalent scaffold amongst LpxC inhibitors. It is anticipated that insights gained from this study would be instrumental for the future design and discovery of LpxC inhibitors.

## Declaration

### Conflict of interests

The authors declare that no competing interests exist.

### Acknowledgments

This research project is supported by Mahidol University (Basic Research Fund: fiscal year 2022) and Office of the Permanent Secretary, Ministry of Higher Education, Science, Research and Innovation (grant no. RGNS 64-146) and the National Research Council of Thailand and Mahidol University (NRCT5-RSA63015-17).

## Supplementary Material

Supplementary information

## Figures and Tables

**Table 1:**
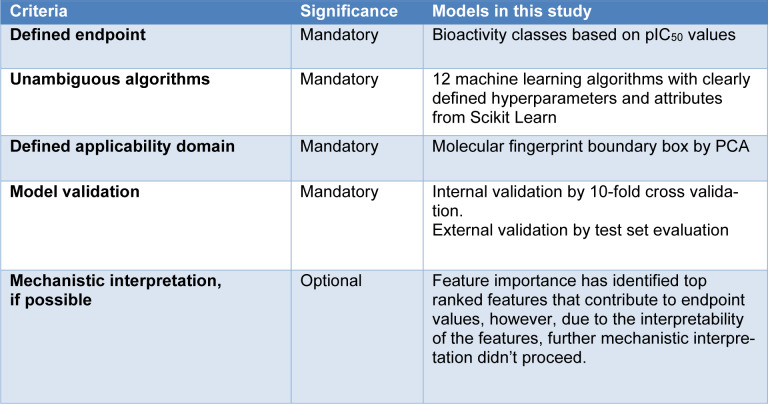
Robustness of the models according to OECD criteria

**Table 2:**
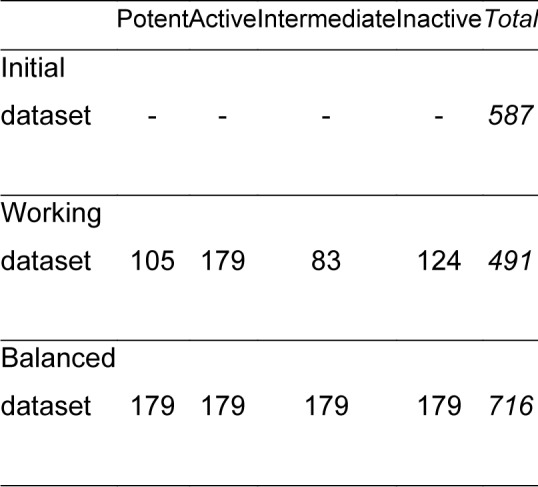
Summary of the dataset used for predicting the activity of LpxC inhibitors

**Table 3:**
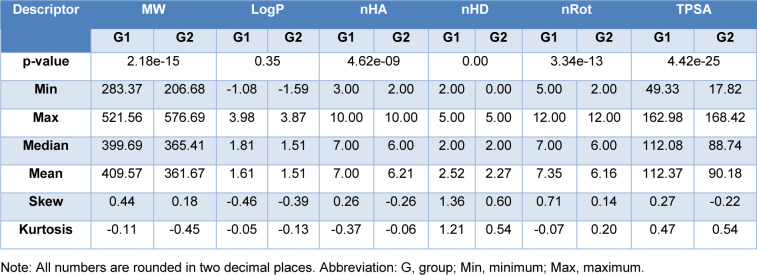
Exploratory data analysis of six drug-likeness descriptors and comparison between group1 and group2 molecules of LpxC inhibitors

**Table 4:**
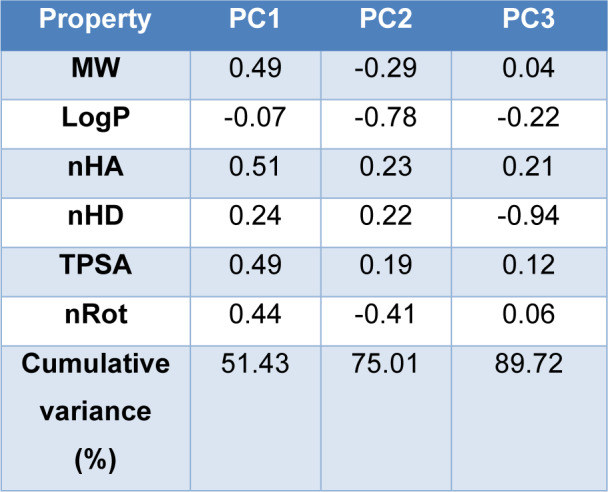
Eigenvalues of the six properties in PCA analysis

**Table 5:**
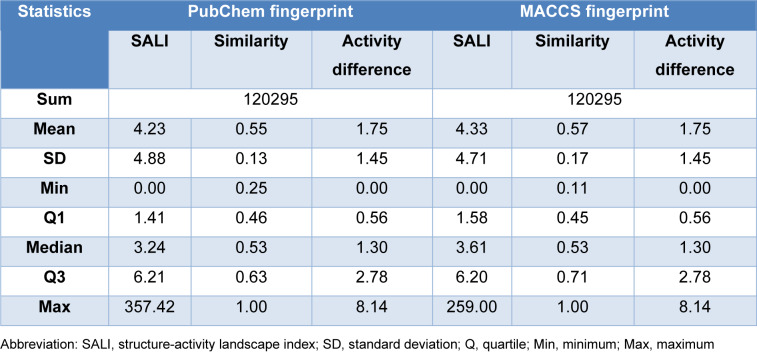
Statistics of SAS map

**Table 6:**
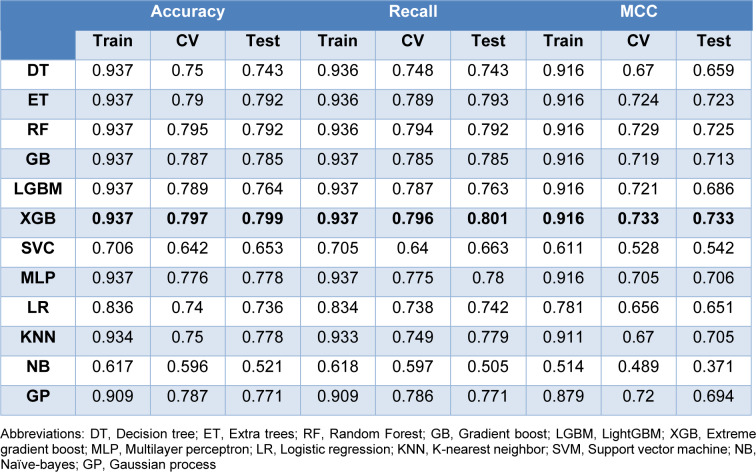
Performance metrics for model 1. Model 1 incorporates PubChem fingerprints (variance threshold=0.10, correlation threshold = 0.95, random state = 42)

**Table 7:**
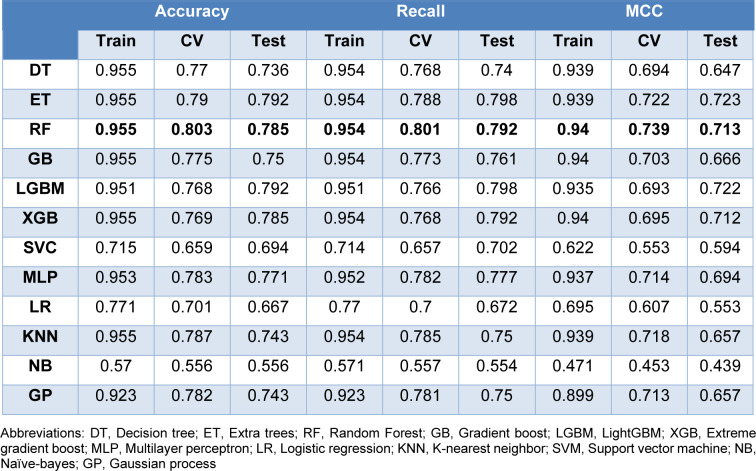
Performance metrics for model 2. Model 2 incorporates MACCS fingerprints (variance threshold=0.10, correlation threshold = 0.95, random state = 42).

**Table 8:**
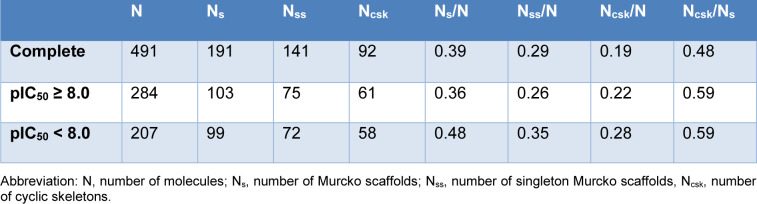
Scaffold diversity analysis for LpxC inhibitors
