# Supplementary material for: Machine learning approaches to study the structure-activity relationships of LpxC inhibitors
Source: EXCLI J. 2023 Sep 5;22:975–91. doi: 10.17179/excli2023-6356 (PMC10630528; doi:10.17179/excli2023-6356)
Supplement: Supplementary information [file EXCLI-22-975-s-001.pdf]

EXCLI Journal 2023;22:975-991 – ISSN 1611-2156
Received: July 15, 2023, accepted: September 01, 2023, published: September 05, 2023

Supplementary information to:

Original article:

MACHINE LEARNING APPROACHES TO STUDY THE STRUCTURE-ACTIVITY RELATIONSHIPS OF LpxC INHIBITORS

Tianshi Yu[1], Li Chuin Chong[2], Chanin Nantasenamat[3], Nuttapat Anuwongcharoen[1], Theeraphon Piacham[4,*]

[1] Center of Data Mining and Biomedical Informatics, Faculty of Medical Technology, Mahidol University, Bangkok, Thailand
[2] Beykoz Institute of Life Sciences and Biotechnology, Bezmialem Vakif University, Beykoz, Istanbul, Türkiye
[3] Streamlit Open Source, Snowflake Inc., San Mateo, California 94402, United States
[4] Department of Clinical Microbiology and Applied Technology, Faculty of Medical Technology, Mahidol University, Bangkok, Thailand

* **Corresponding author:** Theeraphon Piacham, Department of Clinical Microbiology and Applied Technology, Faculty of Medical Technology, Mahidol University, Bangkok, Thailand, Phone: +66 2 441 4371; Fax: +66 2 441 4380,
E-mail: theeraphon.pia@mahidol.ac.th

https://dx.doi.org/10.17179/excli2023-6356

This is an Open Access article distributed under the terms of the Creative Commons Attribution License (http://creativecommons.org/licenses/by/4.0/).

EXCLI Journal 2023;22:975-991 – ISSN 1611-2156
Received: July 15, 2023, accepted: September 01, 2023, published: September 05, 2023

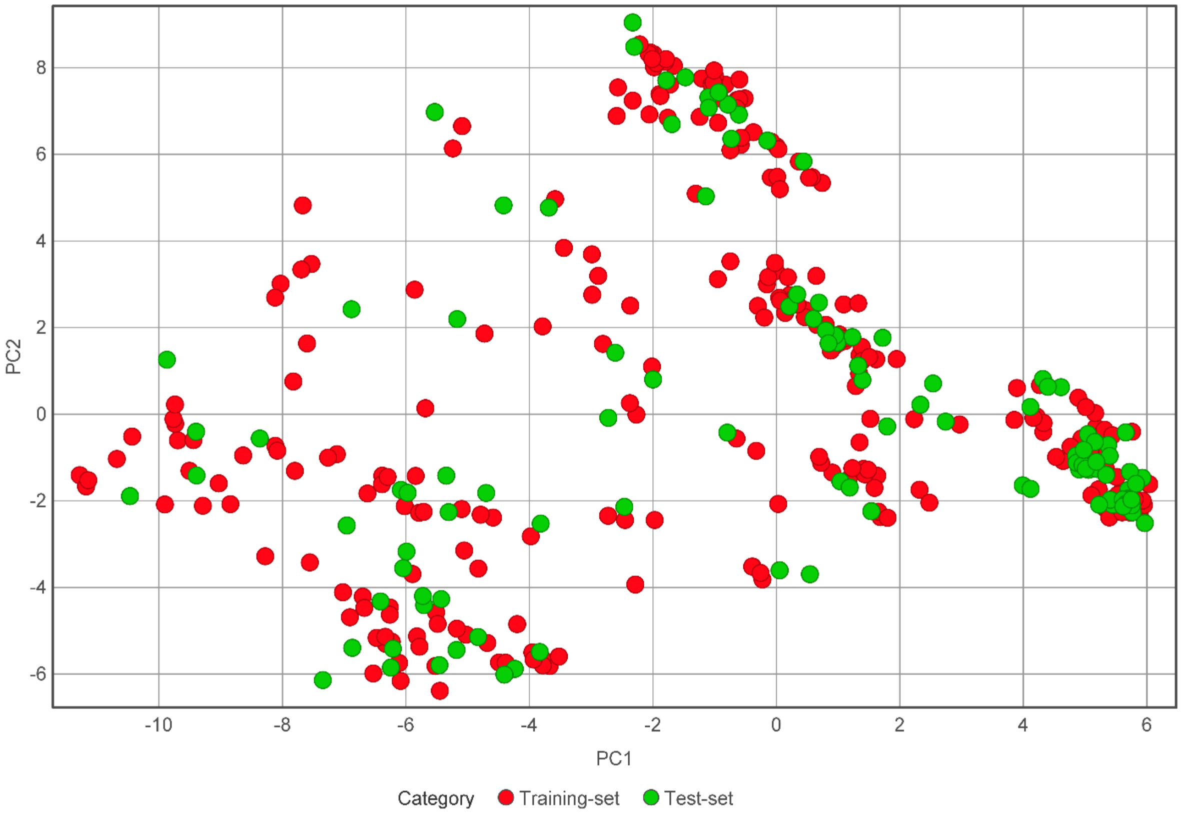

Supplementary Figure 1A: Applicability domain illustration of models using PubChem fingerprint

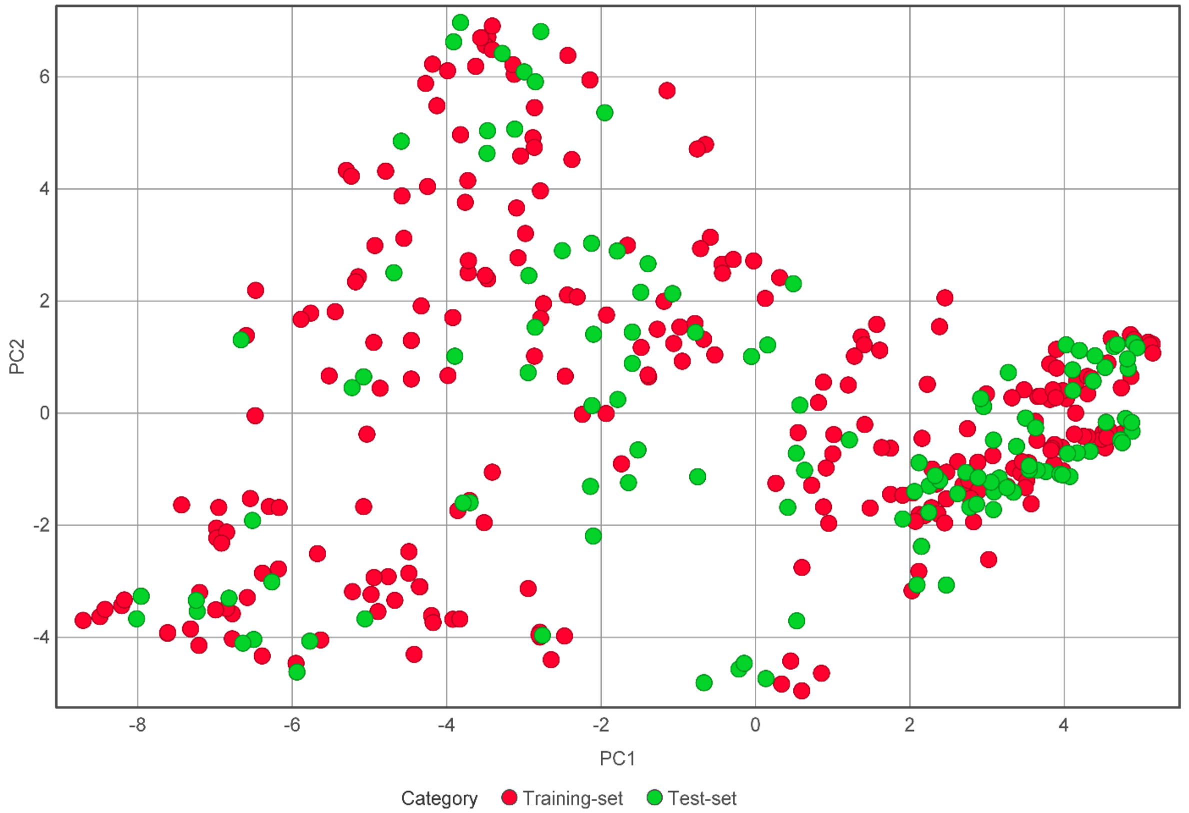

Supplementary Figure 1B: Applicability domain illustration of models using MACCS fingerprint
